# Correction: Pathological mechanism and antisense oligonucleotide-mediated rescue of a non-coding variant suppressing factor 9 RNA biogenesis leading to hemophilia B

**DOI:** 10.1371/journal.pgen.1009345

**Published:** 2021-01-28

**Authors:** Simon Krooss, Sonja Werwitzke, Johannes Kopp, Alice Rovai, Dirk Varnholt, Amelie S. Wachs, Aurelie Goyenvalle, Annemieke Aartsma-Rus, Michael Ott, Andreas Tiede, Jörg Langemeier, Jens Bohne

The eighth author’s name is spelled incorrectly. The correct name is: Annemieke Aartsma-Rus
