# Supplementary material for: Pathological mechanism and antisense oligonucleotide-mediated rescue of a non-coding variant suppressing factor 9 RNA biogenesis leading to hemophilia B
Source: PLoS Genet. 2020 Apr 8;16(4):e1008690. doi: 10.1371/journal.pgen.1008690 (PMC7141619; doi:10.1371/journal.pgen.1008690)
Supplement: S1 Fig — (PDF) [file pgen.1008690.s001.pdf]

Figure S1

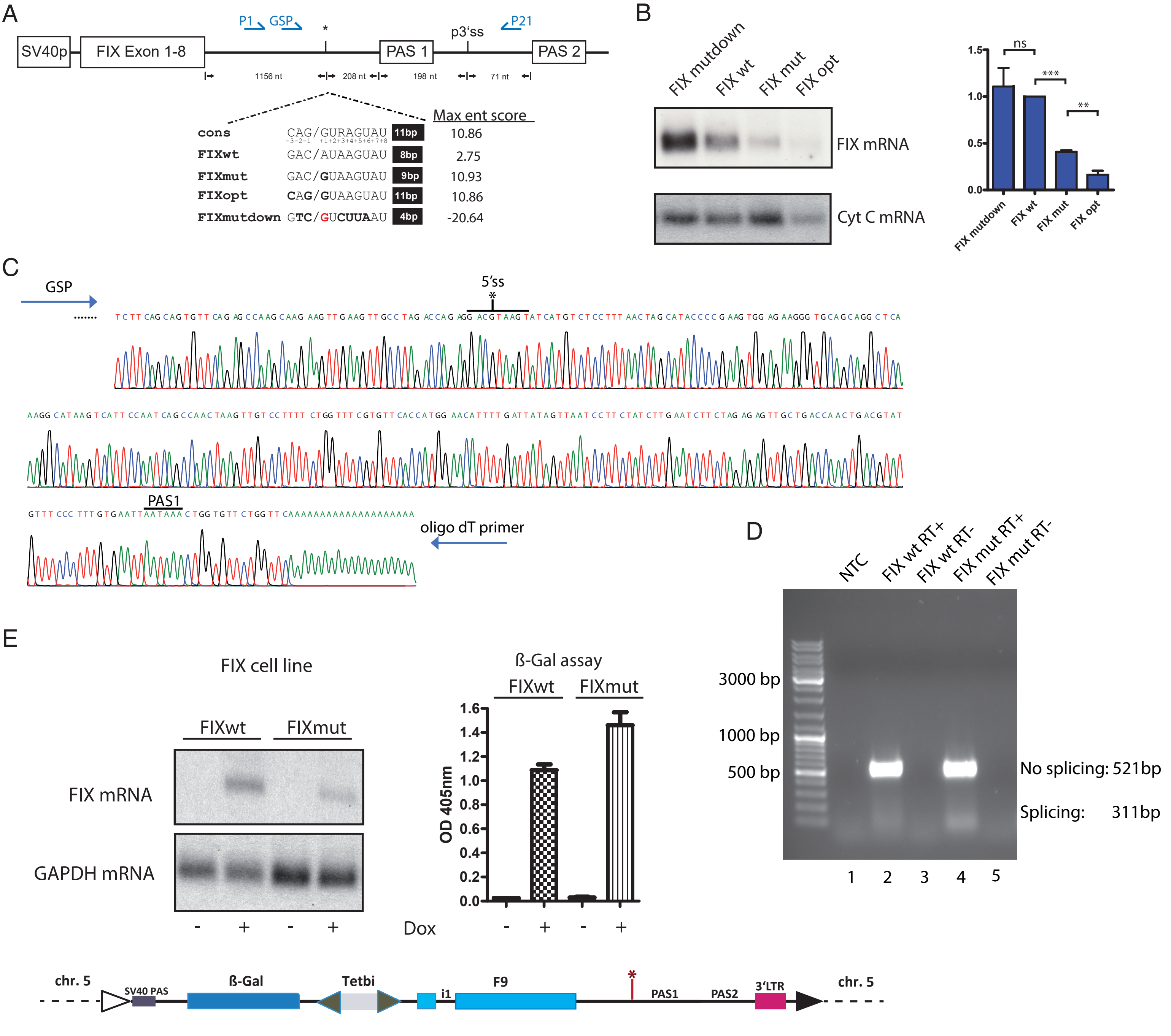

(A) The minigene from Fig. 1A is shown amended with the construct mutdown carrying the mutated base (red, bold) and additional mutations. This constructs allows only 4 interactions with U1snRNA. Also the Max ent score turn negative indicating that the site is not recognized by U1snRNP despite the GU dinucleotide. The primer pairs used in (C) and (D) are indicated by blue arrows. (B) Left panel: northern blot of total RNA from transfected 293T cells. The blot was re-hybrized with probe for ctyochrome C as a loading control. The right panel shows the quantification from three independent experiment by phoshoimager. An unpaired student's T-test was performed (ns, non-significant; *** p<0.0001; ** p<0.0017). (C) Detailed strategy of the 3'RACE with the location of the gene-specific primer in respect to the mutation. (D) Agarose gel analysis of RT_PCRs on RNA derived from F9 transfected Huh-7 cells using the primer pair indicated in (A). An RT reaction omitting RT enzyme was used as a control as well as a non-template control (NTC). The expected products are indicated on the right and the molecular marker on the left. (E) Depiction of the integrated Tetbi construct expressing beta-Gal and our F9 minigene. The black and white triangles represent the Flip sites used for recombinase- mediated cassette exchange on this pre-tagged locus on chromosome 5 [43]. Left below a Northern blot using total RNA from uninduced and dox-induced cells is shown. Here the cells express the reverse Tet-transactivator, thus we used a Tet-on system. On the right a β-gal assay from three independent experiments is presented.
