# Supplementary material for: Pathological mechanism and antisense oligonucleotide-mediated rescue of a non-coding variant suppressing factor 9 RNA biogenesis leading to hemophilia B
Source: PLoS Genet. 2020 Apr 8;16(4):e1008690. doi: 10.1371/journal.pgen.1008690 (PMC7141619; doi:10.1371/journal.pgen.1008690)
Supplement: S2 Fig — (PDF) [file pgen.1008690.s002.pdf]

Figure S2

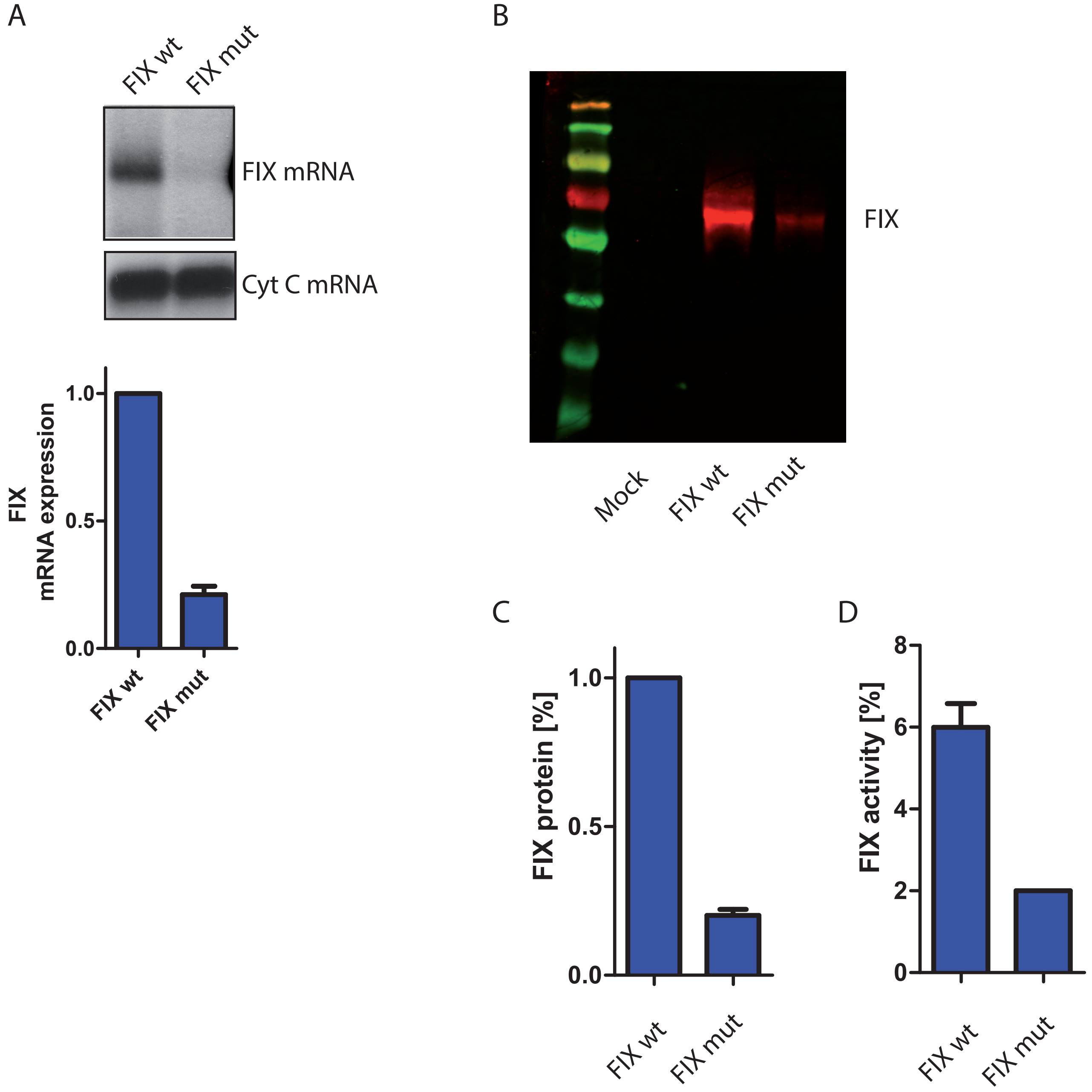

(A) RNA from transfected Huh7 cells was analyzed by Northern blot. The blot was re-probed with a probe directed against ctyochrome C RNA. The panel below shows the quantification of three independent experiments by phosphoimager. The wild type was set to 1. (B) Immunoblot using a Licor imager. The supernatant of transfected Huh7 cells was analyzed. (C) Quantification of the immunoblot from three independent experiments using the licor software. (D) The same supernatants were analyzed for their coagulation activity as in Fig. 3. r

Supplementary Figure S2; Krooss et al., 2019
