# Supplementary material for: Pathological mechanism and antisense oligonucleotide-mediated rescue of a non-coding variant suppressing factor 9 RNA biogenesis leading to hemophilia B
Source: PLoS Genet. 2020 Apr 8;16(4):e1008690. doi: 10.1371/journal.pgen.1008690 (PMC7141619; doi:10.1371/journal.pgen.1008690)
Supplement: S3 Fig — (PDF) [file pgen.1008690.s003.pdf]

Figure S3

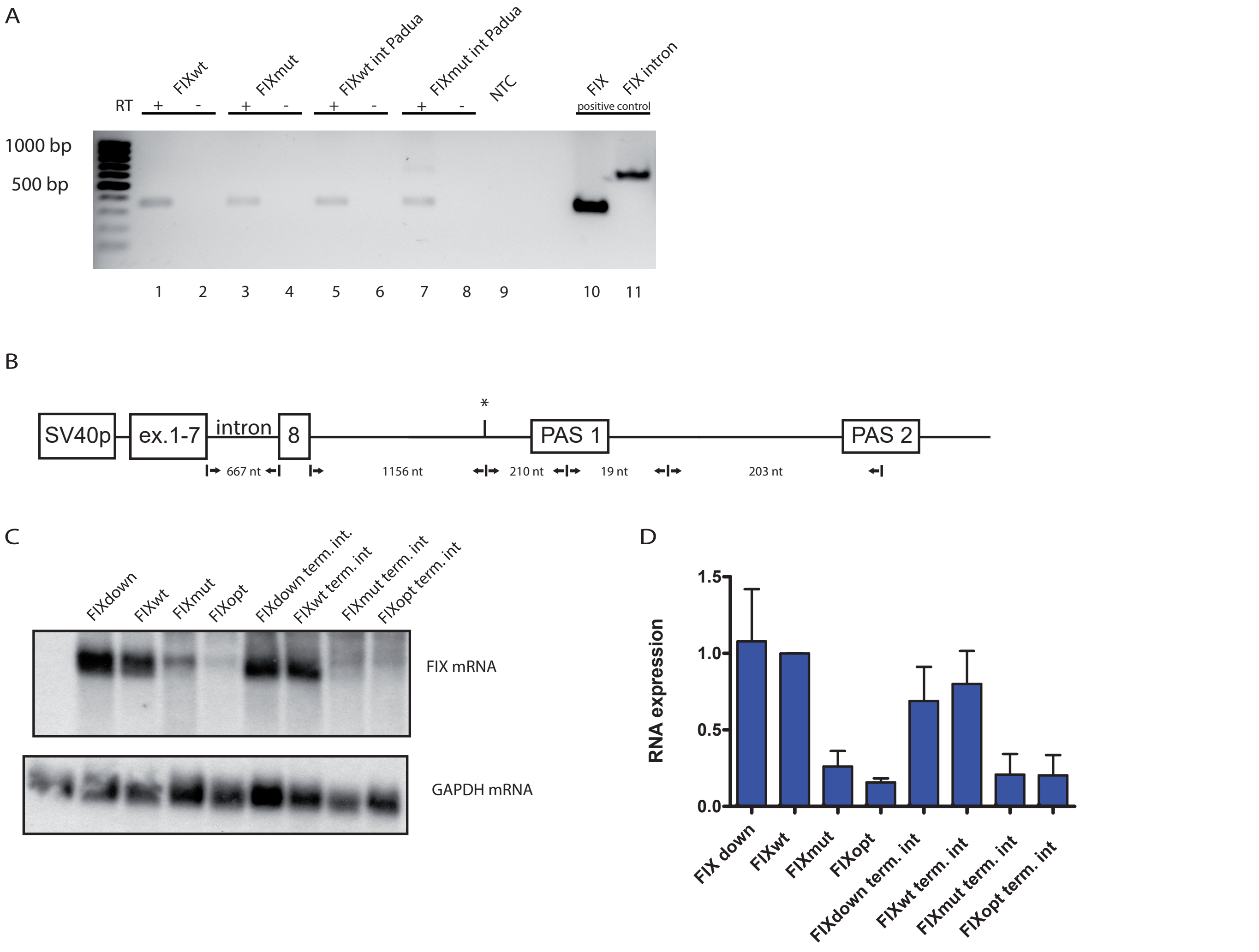

(A) RNA from transfected 293 cells was reverse transcribed with primers flanking intron 1. Products were visualized by gel electrophoresis and EtBr staining. Lane 10 and 11 represent plasmid controls. (B) Depiction of the FIX minigene harboring the terminal intron at its authentic position. (C) Northern blot using total RNA obtained from transient transfection of HEK293T cells. FIX RNA and GAPDH (loading control) were detected as described in the manuscript. (D) Quantification of the RNA data in (C). Three independent experiments were performed.

Supplementary Figure S3; Krooss et al., 2019
