# Supplementary material for: Pathological mechanism and antisense oligonucleotide-mediated rescue of a non-coding variant suppressing factor 9 RNA biogenesis leading to hemophilia B
Source: PLoS Genet. 2020 Apr 8;16(4):e1008690. doi: 10.1371/journal.pgen.1008690 (PMC7141619; doi:10.1371/journal.pgen.1008690)
Supplement: S4 Fig — (PDF) [file pgen.1008690.s004.pdf]

Supplementary Figure S4

A

Both, FIX wt and FIX mut 3’UTR sequences surrounding the mutation were employed for miRDB analysis.

Input sequence (mutated 3’UTR):

GAATTCAACAGTGTGTCTTCAGCAGTGTTCAGAGCCAAGCAAGAAGTTGAAGTTGCCTAGACCAGAGGAC**G**TAAGTATCATGTCTCCTTTAACTAGCATACCCCGAAGTGGAGAAGGGTGCAGCAGGCTCAAAG
GCATAAGTCATTCCAATCAGCCAACTAAGTTGTCCTTTTCTGGTTTCGTGTTCACCATGGAACATTTTGATTATAGTTAATCCTTCTATCTTGAATCTTCTAGAGAGTTGCTGACCAACTGACGTATGTTTCCCTTTGT
GAATT**AATAAA**

3 miRNA binding sites were predicted in the given sequence

---

1)

**miRNA Name**	hsa-miR-4261	**miRNA Sequence**	AGGAAACAGGGACCCA
**Target Score**	78	**Seed Location**	261
**Target Length**	284		

Custom Target Sequence

```
  1 GAATTCAACA GTGTGTCTTC AGCAGTGTTC AGAGCCAAGC AAGAAGTTGA AGTTGCCTAG
 61 ACCAGAGGAC GTAAGTATCA TGTCTCCTTT AACTAGCATA CCCCGAAGTG GAGAAGGGTG
121 CAGCAGGCTC AAAGGCATAA GTCATTCCAA TCAGCCAACT AAGTTGTCCT TTTCTGGTTT
181 CGTGTTCACC ATGGAACATT TTGATTATAG TTAATCCTTC TATCTTGAAT CTTCTAGAGA
241 GTTGCTGACC AACTGACGTA TGTTTCCCTT TGTGAATTAA TAAA
```

2)

**miRNA Name**	hsa-miR-6852-3p	**miRNA Sequence**	UGUCCUCUGUUCCUCAG
**Target Score**	65	**Seed Location**	64
**Target Length**	284		

Custom Target Sequence

```
  1 GAATTCAACA GTGTGTCTTC AGCAGTGTTC AGAGCCAAGC AAGAAGTTGA AGTTGCCTAG
 61 ACCAGAGGAC GTAAGTATCA TGTCTCCTTT AACTAGCATA CCCCGAAGTG GAGAAGGGTG
121 CAGCAGGCTC AAAGGCATAA GTCATTCCAA TCAGCCAACT AAGTTGTCCT TTTCTGGTTT
181 CGTGTTCACC ATGGAACATT TTGATTATAG TTAATCCTTC TATCTTGAAT CTTCTAGAGA
241 GTTGCTGACC AACTGACGTA TGTTTCCCTT TGTGAATTAA TAAA
```

3)

MicroRNA and Target Gene Description:

**miRNA Name**	hsa-miR-517-5p	**miRNA Sequence**	CCUCUAGAUGGAAGCACUGUCU
**Previous Name**	hsa-miR-517*		
**Target Score**	61	**Seed Location**	233
**Target Length**	284		

Custom Target Sequence

```
  1 GAATTCAACA GTGTGTCTTC AGCAGTGTTC AGAGCCAAGC AAGAAGTTGA AGTTGCCTAG
 61 ACCAGAGGAC GTAAGTATCA TGTCTCCTTT AACTAGCATA CCCCGAAGTG GAGAAGGGTG
121 CAGCAGGCTC AAAGGCATAA GTCATTCCAA TCAGCCAACT AAGTTGTCCT TTTCTGGTTT
181 CGTGTTCACC ATGGAACATT TTGATTATAG TTAATCCTTC TATCTTGAAT CTTCTAGAGA
241 GTTGCTGACC AACTGACGTA TGTTTCCCTT TGTGAATTAA TAAA
```

Input sequence (wild type 3’UTR):

GAATTCAACAGTGTGTCTTCAGCAGTGTTCAGAGCCAAGCAAGAAGTTGAAGTTGCCTAGACCAGAGGACATAAGTATCATGTCTCCTTTAACTAGCATACCCCGAAGTGGAGAAGGGTGCAGCAGGCTCAAAG GCATAAGTCATTCCAATCAGCCAACTAAGTTGTCCTTTTCTGGTTTCGTGTTCACCATGGAACATTTTGATTATAGTTAATCCTTCTATCTTGAATCTTCTAGAGAGTTGCTGACCAACTGACGTATGTTTCCCTTTGT GAATT**AATAAA**

3 miRNA binding sites were predicted in the given sequence

1)
MicroRNA and Target Gene Description:

**miRNA Name**	hsa-miR-4261	**miRNA Sequence**	AGGAAACAGGGACCCA
**Target Score**	78	**Seed Location**	261
**Target Length**	284		

Custom Target Sequence

```
  1 GAATTCAACA GTGTGTCTTC AGCAGTGTTC AGAGCCAAGC AAGAAGTTGA AGTTGCCTAG
 61 ACCAGAGGAC ATAAGTATCA TGTCTCCTTT AACTAGCATA CCCCGAAGTG GAGAAGGGTG
121 CAGCAGGCTC AAAGGCATAA GTCATTCCAA TCAGCCAACT AAGTTGTCCT TTTCTGGTTT
181 CGTGTTCACC ATGGAACATT TTGATTATAG TTAATCCTTC TATCTTGAAT CTTCTAGAGA
241 GTTGCTGACC AACTGACGTA TGTTTCCCTT TGTGAATTAA TAAA
```

2)
MicroRNA and Target Gene Description:

**miRNA Name**	hsa-miR-6852-3p	**miRNA Sequence**	UGUCCUCUGUUCCUCAG
**Target Score**	73	**Seed Location**	64
**Target Length**	284		

Custom Target Sequence

```
  1 GAATTCAACA GTGTGTCTTC AGCAGTGTTC AGAGCCAAGC AAGAAGTTGA AGTTGCCTAG
 61 ACCAGAGGAC ATAAGTATCA TGTCTCCTTT AACTAGCATA CCCCGAAGTG GAGAAGGGTG
121 CAGCAGGCTC AAAGGCATAA GTCATTCCAA TCAGCCAACT AAGTTGTCCT TTTCTGGTTT
181 CGTGTTCACC ATGGAACATT TTGATTATAG TTAATCCTTC TATCTTGAAT CTTCTAGAGA
241 GTTGCTGACC AACTGACGTA TGTTTCCCTT TGTGAATTAA TAAA
```

3)
MicroRNA and Target Gene Description:

**miRNA Name**	hsa-miR-517-5p	**miRNA Sequence**	CCUCUAGAUGGAAGCACUGUCU
**Previous Name**	hsa-miR-517*		
**Target Score**	61	**Seed Location**	233
**Target Length**	284		

Custom Target Sequence

```
  1 GAATTCAACA GTGTGTCTTC AGCAGTGTTC AGAGCCAAGC AAGAAGTTGA AGTTGCCTAG
 61 ACCAGAGGAC ATAAGTATCA TGTCTCCTTT AACTAGCATA CCCCGAAGTG GAGAAGGGTG
121 CAGCAGGCTC AAAGGCATAA GTCATTCCAA TCAGCCAACT AAGTTGTCCT TTTCTGGTTT
181 CGTGTTCACC ATGGAACATT TTGATTATAG TTAATCCTTC TATCTTGAAT CTTCTAGAGA
241 GTTGCTGACC AACTGACGTA TGTTTCCCTT TGTGAATTAA TAAA
```

B

Off-Target screening of U7 Sequence: CCTAGACCAGAGGACGTAAGTATCATGTCT

BLAT Analysis from UCSC Genome Browser:

Alignment of YourSeq and chrX:139563213-139563242

Click on links in the frame to the left to navigate through the alignment. Matching bases in cDNA and genomic sequences are colored blue and capitalized. Light blue bases mark the boundaries of gaps in either sequence (often splice sites).

cDNA YourSeq

```
CCTAGACCAG AGGACgTAAG TATCATGTCT
```

Genomic chrX :

```
agaaccgttc gtttgcaatc tacagctagt agagactttg aggaagaatt  139563162
caacagtgtg tcttcagcag tgttcagagc caagcaagaa gttgaagttg  139563212
CCTAGACCAG AGGACaTAAG TATCATGTCT cctttaacta gcataccccg  139563262
aagtggagaa gggtgcagca ggctcaaagg cataagtcat tccaatcagc  139563312
caactaagtt gtccttttct ggtttcgtgt
```

Side by Side Alignment

```
000000001 cctagaccagaggacgtaagtatcatgtct 000000030
>>>>>>>>> |||||||||||||| ||||||||||||||| >>>>>>>>>
139563213 cctagaccagaggacataagtatcatgtct 139563242
```

**Aligned Blocks with gaps <= 8 bases are merged for this display when only one sequence has a gap, or when gaps in both sequences are of the same size.*

Human BLAT Results

BLAT Search Results

Go back to chr1:11102837-11267747 on the Genome Browser.

Custom track name: blat YourSeq Custom track description: blat on YourSeq

Build a custom track with these results

ACTIONS	QUERY	SCORE	START	END	QSIZE	IDENTITY	CHRO	STRAND	START	END	SPAN
browser details	YourSeq	28	1	30	30	96.7%	X	+	139563213	139563242	30

Supplementary Figure S5; Krooss et al., 2020
