# Supplementary material for: Pathological mechanism and antisense oligonucleotide-mediated rescue of a non-coding variant suppressing factor 9 RNA biogenesis leading to hemophilia B
Source: PLoS Genet. 2020 Apr 8;16(4):e1008690. doi: 10.1371/journal.pgen.1008690 (PMC7141619; doi:10.1371/journal.pgen.1008690)
Supplement: S1 Table — (PDF) [file pgen.1008690.s005.pdf]

Patient	FIX:C(%)	FIX:Ag(%)	Inheritance	Severity	Type	Inhibitors	Country	Comments	Reference
1	<1	<1	Familial	Severe	-		USA	-	Chen et al (1995)
2	2	<1		Moderate	I		USA	-	Vielhaber et al (1993)
3	3	<1		Moderate	I		USA	-	Vielhaber et al (1993)
4	1,30	<1		Moderate	I		USA	-	Vielhaber et al (1993)
5	-	-		Severe	-		USA	-	Vielhaber et al (1993)
6	<1	-		Severe	-		United Kingdom	-	Rowley et al (1995)
7	<1	0.6		Severe	-		United Kingdom	-	Rowley et al (1995)
8	1	-		Moderate	-		United Kingdom	-	Rowley et al (1995)
9	2	-		Moderate	-		United Kingdom	-	Rowley et al (1995)
10	4	-		Moderate	-		United Kingdom	-	Rowley et al (1995)
11	1	10		Moderate	II		United Kingdom	-	Rowley et al (1995)
12	-	-			-			-	Centre B9 (unpublished)
13	-	-			-		USA	-	Ketterling et al (1999)
14	4,50	-		Moderate	-		China	-	Liu et al (2000)
15	2	-		Moderate	-			-	Centre B37 (unpublished)
16	<1	-		Severe	-			-	Centre B14 (unpublished)
17	1	-		Moderate	-			-	Centre B14 (unpublished)
18	-	-		Moderate	-		Italy	-	Belvini et al (2005)
19	<1	-	Familial	Severe	-		Jordan	-	Awidi et al (2011)
20	33.4	-		Mild	-		USA	-	Li et al (2000)
21	<1	-	Familial	Severe	-		Jordan	-	Awidi et al (2011)

Supplementary Table 1
List of all described patients with c.2545A>G mutation adapted from the FIX database (22nd of August 2018). Except for patient 20, all others exhibit either a moderate or severe hemophilia B. The mutation occurs in 5 different geographic regions.
