# Supplementary material for: Pathological mechanism and antisense oligonucleotide-mediated rescue of a non-coding variant suppressing factor 9 RNA biogenesis leading to hemophilia B
Source: PLoS Genet. 2020 Apr 8;16(4):e1008690. doi: 10.1371/journal.pgen.1008690 (PMC7141619; doi:10.1371/journal.pgen.1008690)
Supplement: Attachment — Submitted filename: Kroos et al_Rebuttal_plos genetics_final.doc [file pgen.1008690.s006.doc]

MHH-Institut für Virologie - Carl-Neuberg-Str. 1 - 30625 Hannover

**Prof. Dr. Thomas F. Schulz**

**Institut für Virologie**

[Schulz.Thomas@mh-hannover.de](mailto:Schulz.Thomas@mh-hannover.de)

PD Dr. Jens Bohne

[Bohne.Jens@mh-hannover.de](mailto:Bohne.Jens@mh-hannover.de)

Carl-Neuberg-Straße 1

30625 Hannover

Telefon: 0511 532-4307

Fax: 0511 532-8736

[www.mh-hannover.de](http://www.mh-hannover.de/)

To

Mrs. Alice Musson

Publications Assistant

PLOS Genetics

Hannover, 7^th^ of January 2020

**Rebuttal letter for manuscript PGENETICS-D-19-01514**

**Dear Mrs. Musson**

Thank you for the opportunity to submit a revised version. Please find our point-by-point response to the concerns raised by the reviewers below. We are grateful for the suggestions of the reviewers, which led to a substantial improvement of our manuscript. We hope that our manuscript now merits publication in PLOS genetics.

Sincerely,

Jens Bohne (on behalf of all coauthors)

**Reviewer 1:**

**Major point (1):** *My main concern is the use of several different cell contexts. It seems that HEK 293T, CHO, Huh7, and HeLa cells are all used in different places. It is disappointing that better data was not obtained in the liver-derived Huh7 cells that might be conjectured to be the most relevant to factor 9 biology. It appears that some cell types were better suited for protein assays and others for mRNA assays. The authors must clarify the reasons for using different cell types in the various assays.*

This is a very important point and we apologize for not clarifying this issue in depth in the manuscript. However, there are good reasons to use these different cell lines to assay various aspects of the 3’UTR variant, which are given below and in the manuscript on page 5, line 134; page 6, line 170, page 8, lines 218 to 219, page 9, lines 242 to 244, page 10, lines 275-279. In the end the argument could also be that the pathomechanism of the variant functions via a ubiquitous cellular pathway (U1-dependent suppression of 3’ end processing) and thus, should be similar in various cell types, which we indeed observed.

**293T:** these cells were used initially to pin down the phenotype of the mutation and to optimize the FIX minigenes for higher expression levels. There are characterized by a high transfection efficiency, which made the initial observations possible.

**CHO:** this cell line is also used for industrial production of factor IX for infusion into the patients as stated on page 8, lines 218 to 219. For unknown reasons these cells secret the highest amount of completely modified FIX. Since we sought to reproduce our phenotype also on the level coagulation activity and supernatant from other cell lines failed, we made use of yet another cell line (CHO). In the end, the coagulation phenotype could be best reproduced in the CHO cells.

**Huh7:** we thought it is important to reproduce the phenotype of the mutation in a hepatic cell line. We were successful, but also noticed a lower transfection efficiency in these cells. Other hepatic cell lines tested (e. g. HepG3) had even lower transfection efficiencies. For this reason we did not perform all experiments using Huh7 cells. Although Huh7 cells are designated as well-differenciated hepatocytes they do not express endogenous FIX. Thus, Huh7 cells do not display a strong advantage over the CHO cell line.

**HeLa:** in order to show the direct involvement of U1snRNA in the pathomechanism we chose to use antisense morpholinos to block 5’ splice site recognition by U1snRNA. However, based on earlier experiments only HeLa cells are resilient enough to tolerate the treatment of AMO introduction by nanofection (essentially electroporation). For this reason the experiments involving AMOs, LNAs and the 2OMePS oligos were performed in HeLa cells.

**Minor points**

*p.3 line 82: “At present, the FIX mutation database lists 21 patients from different geographic regions (*[*http://www.factorix.org*](http://www.factorix.org/)*; (7, 8); supplementary table 1)”. I believe “with this variant” should be added to the end of the sentence.*

This has been corrected.

*p.3 line 91: “Again several conserved sequence features the hexameric poly(A) signal (PAS) direct the assembly of the cleavage and polyadenylation (CPA) machinery.” This is confusing and should be re-worked.*

This has been corrected.

*p. 7 line 191: “The intron is efficiently spliced (Fig.192 S3A) and it strongly enhances F9 RNA and protein levels (Fig. 2B, lane 6 and 2C, lane 4).” Is there evidence of an enhancer element in this intron?*

We thank the reviewer for this tip. Indeed there is work from the Kurachi laboratory identifying an enhancer present in F9 intron 1. We now state this on page 7, lines 199-200.

*p. 10 line 269 “This vector change was necessary, since our optimized constructs are highly overexpressed in HeLa cells making rescue experiments difficult (data not shown)” This is a bit concerning. It ties into my general concern about the switching of cell types throughout the paper.*

The higher expression of the optimized constructs made a rescue difficult, since the nuclear ratio of F9 RNA to the antisense oligonucleotide seemed to be unfavorable. The same is true for transfection of the U7 antisense plasmid (Fig. 5B). Here, the molar ratio between the SV40-driven FIX minigenes and the U7 plasmid gave a partial rescue. However, the experiments presented in Fig. 5A, B should only represent a proof-of-concept. The partial rescue of F9 mutant RNA, FIX protein and coagulation activity in CHO cells were performed with optimized CMV-driven constructs. However, the sentence was changed on page 10, line 275-279.

**Reviewer 2:**

**Specific point (1):** *One major concern is that all work was done using transfected, or rarely, integrated minigenes. Minigenes with different architectures gave similar results but still it would be particularly convincing if the results were shown in patient cells if they are available. The results are a proof of principle but given the partial rescues in Fig 5 and the reliance on only minigenes, the results are borderline preliminary.*
We agree with the reviewer, but we unfortunately could not obtain patient material. Of course minigenes exhibit limitations; however, we could reproduce the phenotype of the mutation on all levels of gene expression, up to a reduced coagulation activity in cell culture supernatant derived from cells transfected with mutant minigene. One approach in the future could be the introduction of the point mutation by CRISPR-Cas9 base editing. However, one has to keep in mind that most hepatic cell lines do not express FIX, except a commercial cell line named HepaRG. But even for this cell line the evidence in the literature is thin. Therefore, the minigenes represent a reasonable approach to clarify the pathomechanism of this non-coding variant. To mimic the effect of the mutation on a chromosomal level, we additionally generated cell lines with one copy of the factor IX wildtype or mutated gene at the exact same position within the genome. The stable F9 cell lines behave similar to the transient minigene system.

**Specific point (2):** *The results in Figs 2 and 3 could be combined and there is redundancy with the use of different minigenes.*
From an educational point of view we would like to keep figures 2 and 3 separate. The reviewer is correct regarding a seeming redundancy of the used minigenes. However, in Fig. 2 the optimization of the minigenes is described and Fig. 3 uses the optimized constructs in CHO cells. For this reason we first had to reproduce the mutant phenotype with the indicated construct in this new cell line (Fig. 3A).

**Specific point (3):** *Fig 2D – clarify “supernatant”. Presumbably this is protein from the media but not clear.*
We clarified this in the legend to figure 2D.

**Specific point (4):** *Using and ASO to block U1 is an unusual strategy since there should be an effect on most splicing. However here the results support the contention that the mutation affects U1 binding rather than a microRNA.*
We used antisense oligonucleotides on two occasions in this manuscript. To prove that U1snRNP is responsible for the pathomechanism, we used antisense morpholinos (AMOs) complementary to the 5’ end of U1snRNA. This prevents recognition of 5’ splice sites. And indeed, GAPDH RNA splicing was affected as expected (Fig. 4B). However, the U1-dependent suppression of RNA 3’ end processing was also blocked and the mutant F9 RNA was rescued (Fig. 4B). The usage of AMOs is not unusual as the additional role for U1snRNP in keeping transcript integrity was discovered by AMOs (Kaida et al., 2010).

In a more therapeutically relevant approach, we used antisense oligonucleotides (ASOs) to mask the mutant site and prevent U1snRNP from binding (Fig. 5A). This treatment does not affect splicing, since it is specific for the F9 3’UTR.

**Specific point (5):** *While the rescue in Fig 5 was significant, there was not as strong as effect as one might expect. Why weren’t AMOs rather than 2OMe antisense oligos were used to block the mutant site on the minigene mRNA since the AMOs worked so well on U1 snRNA?*

We chose to use 2OMe antisense oligos since they are clinically more relevant. In the next step we moved to the U7snRNA antisense system to prove that also antisense sequences synthesized in the cell have a rescue activity. In addition, delivery of the AMOs required electroporation rather than transient transfection.

*We are number of ASOS tested? Perhaps there are secondary structures of other features that prevent sufficient interaction of ASOs with the mutant site.*

In fact, we tested only one antisense olilgonucleotide according to bioinformatic prediction and we checked both the sequence surrounding the mutation as well as the ASO itself for RNA secondary structures and found none. Until this point we argue for an unfavorable ratio of F9 RNA and ASO molecules in the nucleus.

**Reviewer 3:**

**Major points:**

*1- The Authors do not completely exclude an effect on splicing. It is essential to prove that the mutation creates a functional 5' splice site (SS) and that its recognition by the spliceosomal component U1 snRNP causes F9 mRNA suppression in the absence of splicing. Specifically, (Page 6 lane from lane 149), the 3’RACE experiments do not exclude that the mutation induces a splicing-dependent removal of the first polyA site. This splicing event might occur through the usage of the upstream mutant-created 5’ss and the cryptic downstream 3’ss. To clarify this aspect, the Authors should perform a RT-PCR experiment with primers located before and after the two sites to see if this region is spliced.*

We are very grateful for these remarks by the reviewer. We performed the suggested experiments with a new primer pair and excluded splicing as a potential pathomechanism. This new data is now presented in supplementary Figure S1C and D and in the text on page 5, line 147 and lines 150-152 and on page 6, lines 156-160.

*More in general this part is confused and difficult to follow and should be completely rewritten (for example fig S1C does not seem to evaluate “readthrough” but only the expression levels).*

We appreciate this valuable suggestion by the reviewer. The whole paragraph was revised on page 6, lines 1649-167. The former supplementary figure S1C (now Fig. S1E) was meant to assess the amount of residual RNA expression of the F9 mutant RNA. The read-through was quantitatively assessed in Fig. 1D.

*2-To have a more direct evidence that U1 is involved in polyA suppression (and not splicing), I feel that the Authors should necessarily perform experiment with suppressor U1s that bind to the WT region as previously done in ref 12 and show that the suppressor U1s reduce expression. This is important as the polyA site and the U1 binding site seems quite distant (208 nucleotides).
In addition, the formal prove that the U1 affect polyA is missing as they just show less transcript and “read through”. The experiments I suggest at point 1 and 2 will be useful to reinforce the main mechanistic conclusions that U1 directly suppresses polyA.*

We thank the reviewer for bringing up this point. We performed the suggested experiments using a transient co-transfection approach in 293T cells. The data is presented in the new supplementary Fig. 4 and in the text on page 9 and 10. We cloned an U1snRNA suppressor mutant, which recognizes the wildtype sequence (Fig. S4A). This led to a decrease in F9 wildtype RNA expression. So, U1 is indeed responsible for the observed phenotype.

*4- Some conclusions are overestimated. In the absence of specific experiments, the conclusions should be toned down. I provide here a list of sentences that, in my opinion, need attention.
- Page 11 lane 303. I do not feel that the experiments performed really prove that the variant affect the nuclear quality control and that a failure of 3’ end processing result in RNA degradation. I suggest to tone down/ explain better in light of the available experimental evidence provided.*

This summation of our data is also based on the current literature regarding the surveillance mechanism exerted by U1, which normally prevents intronic polyadenylation. We explain now in more detail and added two references (now page 11, lines 311-313).

*-Page 12 lane 336. The evidence that the ASO binding to the mutant site induce a switch to the downstream polyA site is not evident from the experiment and should be better explained.*

The reviewer is correct. We toned down the statement and mention now a read-through that is induced by the ASO (now page 12, line 346).

*-Page 5 lane 149. As the mutation is in the non-coding 3’UTR, I do not understand why the splicing removal of the first polyA site should induce NMD.*
As mentioned in the introduction on page 4, a splicing event in a 3’UTR can elicit NMD, if it occurs more than 50 nucleotides downstream of the STOP codon. The rules for NMD are delineated in the given reference (Kurosaki, 2016) and this is one of the reasons why most 3’UTRs are devoid of introns.

**Minor points:**

*- Page 3 lane 88. I think that the snRNPs involved in splicing are five and not six. (U7 is in 3’ end processing of histones)*
We are sorry for this mistake. The reviewer is correct. U1, U2, U4, U5 and U6 are involved in splicing and U3 is part of snoRNA processing and U7 is essential for processing of cell-cycle dependent histone mRNA processing. We have corrected the mistake.

*-The rationale of using the gain of function Padua mutant in the context of the 3’UTR variant is unclear, as it seems simply an additional control.*
The Padua mutation functions in our view as a magnifier. Especially while looking at the coagulation data presented in Fig. 5G, the Padua mutation proved to be very useful, since it increases activity on the enzymatic level, while the phenotype of the mutation persisted.

*-The Authors claim that transcription is not involved? Why increased transcription does not compensate? This is strange.*
It simply means that increased transcription cannot “overrun” the effect of the mutation. In other words, the amount of U1 is not limiting and the suppression is tight. This is also exemplified by the high expressivity of the mutation.  *-Page 4 lane 112. I think that the most appropriate verb is “reduces” and not “impedes” F9 expression.*
With the use of the verb “impede” we intended to stress that the variant leads to a rapid nuclear degradation. Of course, this also reduces the amount of F9 mRNA and we changed the title of this section accordingly.  *-The Authors identified with in silico approaches two polyA sites that were then tested in expression plasmids and the effect of the mutation is on the proximal one. Is this polyA site the most used or the only one used also in vivo in hepatocytes?*
The PAS1 is the only one used in vivo. PAS2 is not used for a yet unknown reason (Krooss et al. under preparation).  *-Fig 1 B and S1B lack of appropriate statistical analysis. Fig 3b lacks quantification.*

The reviewer is correct. For Fig. 1B the standard deviation of three independent experiments is presented. A statistical analysis revealed a highly significant downregulation of the mutant F9 RNA. This is now indicated in the figure and in the legend. The same was done for Fig. 1SB. Importantly, there is no difference between the mutdown and the wildtype FIX minigene.

The ELISA shown in Fig. 3C is more quantitative than the immunoblot in Fig. 3B. We were worried that the signals in the immunoblot might be out of range taken into account the low signal for the CMV wt and mut constructs. The ELISA is able to quantify minimal FIX amounts. As shown in Fig. 3C the ELISA results mirror the FIX amounts of the immunoblot.

According to the new data policy of PLOS Genetics we removed all “data not shown” references. Of course these data could be made available to the reviewers. However, in our opinion they do not warrant another supplementary figure. In all cases they refer to initial experiments to set up our experimental system and to test suitable cell lines or transfection conditions.
