# Supplementary material for: Pathological mechanism and antisense oligonucleotide-mediated rescue of a non-coding variant suppressing factor 9 RNA biogenesis leading to hemophilia B
Source: PLoS Genet. 2020 Apr 8;16(4):e1008690. doi: 10.1371/journal.pgen.1008690 (PMC7141619; doi:10.1371/journal.pgen.1008690)
Supplement: Attachment — Submitted filename: Krooss et al_2nd_Rebuttal_plos genetics_final.doc [file pgen.1008690.s007.doc]

MHH-Institut für Virologie - Carl-Neuberg-Str. 1 - 30625 Hannover

**Prof. Dr. Thomas F. Schulz**

**Institut für Virologie**

[Schulz.Thomas@mh-hannover.de](mailto:Schulz.Thomas@mh-hannover.de)

PD Dr. Jens Bohne

[Bohne.Jens@mh-hannover.de](mailto:Bohne.Jens@mh-hannover.de)

Carl-Neuberg-Straße 1

30625 Hannover

Telefon: 0511 532-4307

Fax: 0511 532-8736

[www.mh-hannover.de](http://www.mh-hannover.de/)

To

Gregory M. Cooper, PhD
Associate Editor
PLOS Genetics

Hannover, 13^th^ of February 2020

**Rebuttal letter for manuscript PGENETICS-D-19-01514**

**Dear Dr. Cooper**

Thank you for the opportunity to submit our minor revised version. Please find our point-by-point response to the remaining concerns raised by the reviewer 3 below. We hope that our manuscript now merits publication in PLOS genetics.

Sincerely,

Jens Bohne (on behalf of all coauthors)

**Reviewer 3:**

**Point (1):** *In my opinion the Authors have now sufficient evidence to prove that the creations of a consensus 5’ss in the 3’ UTR reduces the total amount of mRNA through an U1snRNP-dependent inhibition of polyadenylation as well as to exclude that the variant induces aberrant splicing. The other potential mechanisms the Authors exclude, miRNA , RBPs and NMD, are not experimentally validated (miRNA and RBPs only in silico and NMD just because splicing is not involved). In particular, for NMD the minigene systems in general (and in particular the one used here, which has no introns) are not NMD sensitive. My suggestion is to focus, in the result section, on the experimentally validated mechanism first and then say that the other mechanisms, based on indirect evidence, are very unlikely.*

We agree with the reviewer that a modified order of presenting the possible and the actual mechanism of the mutation would result in more sound manuscript. This has now been changed on pages 6 and 10.

However, we disagree with reviewer that our reporter construct is not NMD sensitive. If the 5’SS would splice to the putative 3’SS (which is part of our minigene) an exon junction complex would assemble, which is more than 50 nts downstream of the F9 STOP codon. In this scenario NMD would be the consequence.

**Point (2):** *The Authors have correctly analyzed the effect of the suppressor U1 on the WT mutant clearly showing that it significantly reduces the amount of mRNA. I believe this is an important and critical experiment that prove the direct involvement of U1 and accordingly it could be inserted in the main figures. (If there is no space one of the two ASO approaches that block the U1 snRNP should go into supplementary). However, I feel that there is a mistake in the data interpretation at page 10 lanes 270-273 concerning the effect of suppressor U1s on the mutant minigene. I do not see an increase in RNA expression after transfection of the suppressor U1 and I do not understand why a “stronger F9 mutant mRNA increase” should be expected in this case. The Authors should note that the suppressor U1 makes a good complementarity also with the mutant sequence through a wobble G-U base pairing, thus I would expect a further reduction in the mRNA. In any case, the suppressor U1 experiment are not designed to block the U1 snRNP and thus this experiment should be presented in a separate result section.*

First of all, we are grateful for the initial suggestion of reviewer 3 to perform the suppressor snRNA experiment. We now present the data as a new Figure 5 and also as a new paragraph on page 9 and 10.

The reviewer is absolutely correct that the suppressor mutant is still able to pair with the mutated sequence. We missed this interpretation and instead focused on the competition between the suppressor and the wild type U1 snRNA. This has now been corrected on page 10.

**Point (3):** *Unfortunately, the half-life experiments do not prove a co-transcriptionally decay of the mutant F9 mRNA. The mutation clearly reduces but not completely disrupt the usage of the PAS, producing a low amount of normal mature F9 mRNA which has, as expected, a normal half-life. Thus, in the absence of more direct measurement (like Chip assay with PolII or nascent nuclear RNA) the conclusions should be better explained/modified accordingly.*
We agree with the reviewer and replaced this sentence with a toned-down statement.

**Point (4):** *The ASO and the U7 for masking the mutated site seem to induce additional bands (Fig 5 B and D) which are considered to originate from the use of downstream PASs. The Authors speculate in discussion that the ASO might prevent binding of a cellular factor. In the absence of more defined prove of their origin and considering that they are using hybrid minigenes, I suggest to be more cautious in their interpretation. I also see another unclear band in mutant untreated.*

The band was only observed once. So far, we do not have a good explanation for it other than the usage of poly(A) site downstream. Regarding the discussion: we deleted the sentence in which we speculated that the ASO prevents binding of a cellular factor.

According to the new data policy of PLOS Genetics we removed all “data not shown” references. Of course these data could be made available to the reviewers. However, in our opinion they do not warrant another supplementary figure. In all cases they refer to initial experiments to set up our experimental system and to test suitable cell lines or transfection conditions.
